# Effects of Microglial Activation and Polarization on Brain Injury After Stroke

**DOI:** 10.3389/fneur.2021.620948

**Published:** 2021-07-01

**Authors:** Rui Dong, Renxuan Huang, Jiaoqi Wang, Hongyu Liu, Zhongxin Xu

**Affiliations:** ^1^Department of Neurology, China-Japan Union Hospital of Jilin University, Changchun, China; ^2^Department of Neurosurgery, China-Japan Union Hospital of Jilin University, Changchun, China

**Keywords:** microglia, polarization, stroke, MCAO, phenotype

## Abstract

Stroke is one of the most common causes of death worldwide. The subsequent development of neuroinflammation and brain edema dramatically increases the risks associated with stroke, leading to a substantial increase in mortality. Although considerable progress has been made in improving cerebral perfusion in the acute phase of stroke, effective treatment options for the subacute and chronic phases associated with cerebral infarction are limited. Microglia, the innate immune cells of the central nervous system (CNS), can be activated and polarized to take on different phenotypes in response to stimulations associated with stroke, including pro-inflammatory and anti-inflammatory phenotypes, which affect the prognosis of stroke. Therefore, investigation of the activation and polarizing mechanisms of microglia plays a critical role in treating stroke. The aim of this article was to investigate the significance of microglial phenotype regulation in stroke treatment by summarizing the activation, polarizing mechanisms, and general microglia characteristics.

## Introduction

Recent data from the World Health organization (WHO) shows that stroke is a leading cause of death worldwide (1–4). Post-ischemic neuroinflammation and cerebral edema secondary to stroke aggravate the damage caused by stroke to varying degrees (5, 6). Therefore, improvement in therapeutic protocols targeting the regulation of the cerebral microenvironment during stroke, and the repair of neural and tissue damage after stroke, are essential.

Microglia, the resident immune cells in CNS, continuously monitor the brain parenchyma. Microglia can become activated and switch their phenotype in response to changes in the local CNS microenvironment. They can have either a pro- or anti-inflammatory role and influence the prognosis of ischemic stroke (7–9). The M1 phenotype can release cytotoxic factors, such as inflammatory cytokines and nitric oxide (NO). M2-type microglia secrete anti-inflammatory factors, remove cell debris, promote angiogenesis, and promote the repair of injured peripheral nerves (10). Microglia, as an integral part of the immune system, produce inflammatory factors, alter the permeability of the blood-brain barrier (BBB), phagocytose vascular endothelial cells, and lead to BBB breakdown after stroke, which can result in reduced brain recovery following infarction. M1-type microglia also remove necrotic tissue, which promotes neurogenesis, secrete anti-inflammatory factors that regulate inflammation, and produce other neuroprotective effects (11–14). Thus, microglia have a “double-edged sword” function to maintain the homeostasis of the CNS microenvironment after stroke through two opposed pathophysiological effects, both of which play essential roles in recovery after brain injury. Therefore, studying the activation and polarization mechanisms of microglia and exploring possible means of regulating the M1/M2 phenotype transition might provide new ways to improve brain injury treatment after stroke.

This review summarizes the mechanisms associated with microglial activation and polarization, as well as the impact of microglia on stroke prognosis. Also, the therapeutic directions for recovery after a brain injury caused by stroke are discussed. We theorize that limiting microglial hyperactivation, inhibition of the M1 phenotype, and promoting the M2 phenotype through a combination of endogenous mechanisms and exogenous drugs would be beneficial for repairing brain injury following stroke. However, specific mechanisms need further investigation.

## Features of Microglia

Ginhoux has reported that microglia are derived from yolk sac macrophages before embryonic day eight in mice, and contribute to primitive hematopoiesis (15). The microglial stages progress from embryonic to early postnatal then adult CNS (16). Under different conditions of activation, microglia of the developing can display different morphologies (17). Microglia are activated in the embryo and early postnatal brains and display a “generic macrophage”-like and mobile amoeboid morphology, while adult microglia exhibit characteristic ramified extensions that survey the surrounding areas (18).

Early after Middle Cerebral Artery Occlusion (MCAO), activated microglia were observed to be concentrated in the lesion area, and fewer microglia were located inside the ischemic center (19). Over time, the number of microglia in the ischemic center gradually increased, with round and amoeboid characteristics as the predominant morphologies. Highly branched microglia were observed at the borders of the ischemic region during reperfusion for up to 22 h (20). So it is clearly to see that microglia have specific region- and time- dependent features.

Moreover, during embryonic development, the phenotypes and functions of microglia are similar to those of yolk sac macrophages (15). Both cell types share the common characteristics of performing surveillance of the immunological microenvironment, mediating inflammation, and removing debris from dead cells (21). However, these two cells may play different roles during brain injury and can respond in opposite ways to acute inflammatory stimuli (22). According to the results from Yamasaki et al., microglia can protect the injured brain while macrophages concomitantly damage the brain under the same circumstances (21), which may be related to different types of gene expression patterns.

## Activation and Polarization of Microglia

### Microglial Activation and Function

Microglia are the first line of defense against brain injury that takes place following stroke. They respond rapidly to alterations in the brain microenvironment. Early after stroke onset, microglia become activated rapidly, initiate migration, and activate downstream cell signaling (23, 24). Microglia activation is mainly characterized by changes in morphology, phagocytosis, migration to the injured area, and secretion of cytokines and oxidative metabolites such as NO and reactive oxygen species (ROS) (25), the excessive expression of which can lead to neuroinflammation after brain injury (26). In several pathological conditions, including exposure to lipopolysaccharide (LPS), inflammatory factors, hypoxia and other injury- related moleculars, microglia become over activated, and could be induced to exhibit a pro-inflammatory phenotype, where numerous pro-inflammatory cytokines are replicated, leading to neuronal cell damage (27). In fact, not only do microglia aggravate the damage that occurs in the brain and inhibit recovery, but they also remove necrotic cells and debris that promotes nerve regeneration (14). Their phagocytic functions can remove tissue fragments and foreign substances, however, the hyperactivation of microglia and mobilized macrophages caused by stroke reduces their clearance capacity, which could eventually result in damage to neurons and breakdown of the BBB (11, 12).

It's already known that stimulation of microglia with amyloid-β peptide (Aβ) activates transcription factors (e.g., nuclear factor kappa-B, NF-κB) involved in the expression of pro-inflammatory genes (28). On the other hand, nerve growth factor (NGF) promotes TrkA-mediated microglial phagocytosis of Aβ and induces the degradation of Aβ. NGF counteracts the pro-inflammatory activation induced by exposure to Aβ (29). NADPH oxidase (NOX) is a multi-subunit enzyme that produces peroxides and ROS in microglia. The inhibition of NOX significantly reduces pro-inflammatory activation of microglia and produces anti-inflammatory effects (30, 31). Recently, evidence has suggested that MCAO and OGD can induce biphasic microglia activation (32, 33). After ischemia occurs, microglia become activated, and produce detrimental and neuroprotective mediators (34). Thus, microglia carry out dual roles in determining the prognosis of brain injury.

### Microglial Polarization and Function

Microglial polarization follows two pathways, the classical activation (M1) pathway and the alternate activation (M2) pathway. When the M1 phenotype is induced by exposure to LPS, microglia exhibit destructive effects in the CNS. However, the M2 phenotype, which can be induced by IL-4, exhibits neuroprotective effects (14, 35). The M1 phenotype is a pro-inflammatory cell state that releases inflammatory cytokines, including tumor necrosis factor-α (TNF-α), interleukin 6 (IL-6), interleukin 1β (II-1β), NO, and so on. The M1 phenotype is also associated with increased production of ROS, the synthesis of extracellular matrix protein hydrolases (MMP3 and MMP9), and the cell surface marker of M1 phenotype, CD68 and CD16/32 (36–38). M2 microglia express an anti-inflammatory state in which cells release anti-inflammatory mediators, including cellular interleukin-10 (IL-10), cellular interleukin-4 (IL-4), Arginase-1(Arg1), chitinase-like protein-1 (Ym1), transforming growth factor-β (TGF-β). In addition, the cell surface marker of M2 phenotype, CD206, can promote reductions in inflammation, increased clearance, and enhanced expression of homeostasis-associated genes (24, 35, 39).

LPS is a critical component of the cell wall of gram-negative bacteria that induces the release of pro-inflammatory cytokines, produces neuroinflammatory responses, and promotes M1-type microglial polarization^1^ (40). Lipocalin 2 (LCN2) also promotes microglial M1-type activation. The expression of LCN2 in microglia was significantly increased after LPS treatment, indicating increased gene expression related to inflammatory processes in microglia (41).

Microglia and macrophages exhibit a protective M2 phenotype in the early stage after ischemic stroke, then gradually transition to a more classically activated M1 phenotype associated with nerve injury in the later stages after stroke. This transition demonstrates that microglia can exhibit dynamic changes in response to injury at different stages after stroke (23). Therefore, regulation of the microglial M1 and M2 phenotype balance is of great significance for the treatment of stroke.

## Characteristics of Microglial Activation and Polarization in Different Environments

BBB permeability increases with aging, as does the release of pro-inflammatory mediators in the nervous system. When the pro- and anti-inflammatory cytokine levels become unbalanced, this leads to an increased inflammatory state in the brain environment. Also, microglial activation and polarization change with aging (42, 43). It has been reported that decreased expression of chemokines is accompanied by increased microglial activation in the brains of aged rats. Additionally, the treatment of aged rats with chemokines can attenuate microglial activation (13). The data from Shoucai Zhao et al. showed an age-related difference in microglial activation after stroke. Indeed, the young stroke brains had higher IRF4 expression, corresponding to a stronger M2 microglial phenotype, as indicated by an up-regulated membrane CD206 level. In contrast, a stronger M1 phenotype was probably induced by up-regulated expression of IRF5 (44).

Microglia exhibit some differences between sexes. It is widely known that estrogens have anti-inflammatory activity, which may be a determining factor in the gender-dependent manifestations observed in brain lesions. Estrogen and progesterone could have neuroprotective roles in ischemic stroke by regulating the expression of chemokines and enhancing the effect of vascular endothelial growth factor (VEGF) (45). Villa et al. described significant differences in the transcriptomes of microglia from adult male and female that may arise from perinatal exposure to sex steroids (46). They also discovered that microglia from female were neuroprotective because they limited the injury caused by acute focal cerebral ischaemia (46). Bodhankar et al. reported that the expression of microglial M2 markers was higher in female mice than in male mice after ischemic stroke. In contrast, the expression of M1 markers, such as TNF-α and IL-1β, were significantly lower (47).

A recent study revealed that hypothermia has a regulatory effect on brain injury after stroke. Specifically, using a MCAO model, hypothermia decreased the number of M1 microglia and the expression of M1 markers and increased the expression of M2 markers. Thus, hypothermia can transform microglia from the M1 state into the M2 state. This supports the supposition that hypothermia has neuroprotective effects on ischemic stroke (48).

The results discussed above demonstrate that the activation and polarization of microglia differ based on age, sex, time after insult, and location. Microglia also are affected by temperature, and the effects of microglial cell production vary according to their environment. These observations suggest that the types of treatments that are effective and the prognostic results for patients experiencing different types of stroke will be different in clinical settings. These data also provide a range of new ideas to target and design novel treatments for stroke.

## Mechanisms of Microglial Activation and Polarization After Stroke

**Figure 1:**
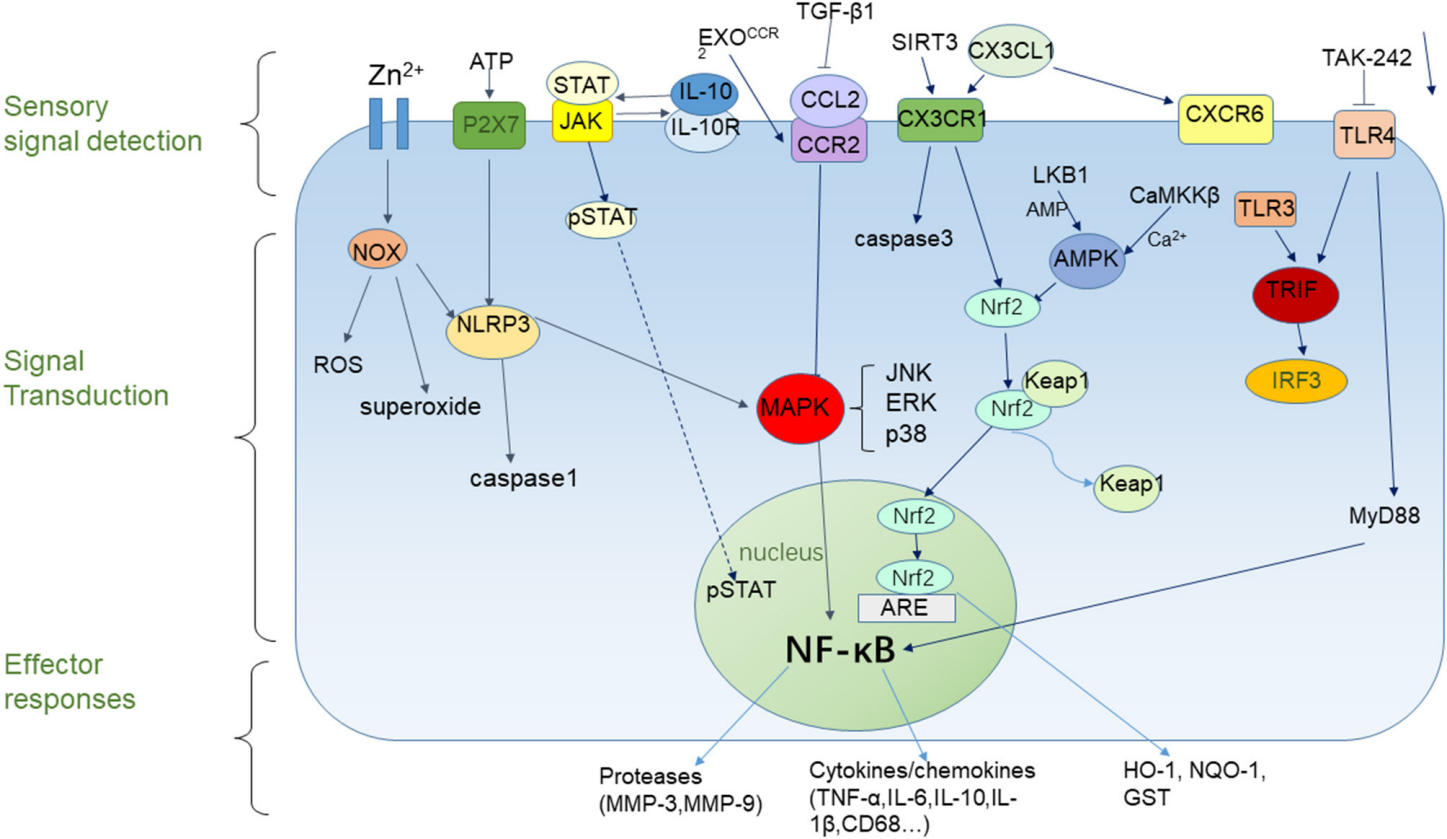
Microglial responses to cerebral ischemia. Microglial responses to cerebral ischemia can be divided into three parts. The sensory component can detect extracellular signals, the signal transduction component can influence gene expression, and the effector component can active pro-inflammatory or anti- inflammatory responses.

Given the importance of microglial responses during brain injury, it is critical to investigate the mechanisms of microglial activation and polarization. This will help to understand the molecular processes that underlie microglial morphological and functional changes caused by changes in the brain microenvironment and also provide direction for developing novel therapies to reduce the damage caused by stroke (Figure 1). According to previous studies, we know that the effects of microglia on cerebral ischemic injury are divided into three processes. First, microglia detect changes in the brain microenvironment after ischemic injury and detect extracellular signals through cell-surface and intracellular receptors. Second, these signals are integrated and transduced, through downstream reactions. Finally, these interactions cause microglia to produce pro-inflammatory or anti-inflammatory effects. Chemokine receptor-ligand pairing, transcription factors, signal transduction, activation of transcription, and other pathways are known to influence microglial activation and polarization through regulation of the activation of the pro-inflammatory transcription factor, NF-κB, which, in turn, regulates cytokine secretion.

NF-κB regulates the transcription of a range of pro-inflammatory factors. After ischemic stroke, NF-κB is activated in activated microglia and translocates from the cytoplasm into the nucleus, which induces the production of inflammatory cytokines and results in secondary brain injury after stroke (49). NF-κB also induces microglia activation and polarization of the M1 phenotype (50). Recently, it has been shown that zinc induces microglial activation through activation of NOX and NF-κB (51). MAPKs are a family of serine/threonine protein kinases that regulate cell proliferation and survival. This family includes JNK, ERK, and p38, which are involved in ischemia-induced neuroinflammation (49). It is evident that MAPK and NF-κB plays an essential role in regulating neuroinflammation after ischemic stroke, as well as the activation and polarization of microglia. Therefore, targeting inhibitors of NF-κB or the activation of pathways that inhibit NF-κB activity might provide a new direction for the treatment of stroke.

### AMPK/Nrf2

Several recent studies have demonstrated that AMP-activated protein kinase (AMPK) and nuclear factor erythroid-2-related factor 2 (Nrf2) play crucial roles in the transition from a pro-inflammatory phenotype in microglia to an anti-inflammatory phenotype (52–54). AMPK is a trimeric serine/threonine kinase with two *in vivo* upstream kinases, including liver kinase B1 (LKB1) and calmodulin-dependent protein kinase kinase β (CaMKKβ). These kinases activate AMPK in AMP-dependent and Ca^2+^-dependent pathways, respectively (55, 56). AMPK, known as a central regulator in the body (57), stimulates energy production by shutting down energy-consuming pathways and phosphorylating antioxidant transcription factors, thereby maintaining metabolic and cellular energy homeostasis (58, 59). Activation of AMPK inhibits NF-κB activation and LPS-mediated pro-inflammatory activation of microglia, promoting M2-type microglial polarization (54). Recently, it has been reported that telmisartan can induce AMPK activation and regulate M2 phenotype polarization in microglia (60).

Nrf2 is a master transcriptional regulator of antioxidant pathways and exerts its anti-inflammatory effects through the activation of antioxidants. The N-terminal domain, Neh2, binds to Kelch-like ECH-associated protein 1 (Keap1) in the cytoplasm. After oxidative stress, the Keap1-Nrf2 complex dissociates, Nrf2 accumulates in the nucleus and binds to antioxidant response elements (ARE) together with small Maf transcription factors. This complex promotes the generation of antioxidant and anti-inflammatory proteins, including heme oxygenase-1 (HO-1), NADPH quinone oxidoreductase-1 (NQO-1), Glutathione S-transferase (GST), and others (61–63). HO-1 has been reported to result from binding of ARE to a dimer, consisting of Nrf2 and activating transcription factor 4 (ATF4) (64). Similar to AMPK, Nrf2 activation suppresses neuroinflammation by inhibiting LPS-induced pro-inflammatory factor expression, and promotes macrophage M2 polarization, resulting in an anti-inflammatory effect (65). Tae et al. found that plumbagin, an activator of the Nrf2/ARE pathway, reduced the area of brain injury in a mouse model of MCAO and reduced the impairment of neurological function (53). The work of Zhang et al. shows that overexpression of SIRT6 in the brain by *in vivo* gene transfer potentiated anti-oxidant NRF2 signaling, reduced oxidative stress and the extent of cerebral I/R-induced brain tissue damage and neurological impairments, while in NRF2 knockout mice these neuroprotective effects were abolished (66). Another result shows that L-F001 can increase the expression levels of the M2 microglia marker CD206 via NRF2 signaling pathways activation *in vitro* (67), which corroborated the protective effect of NRF2 activation.

According to existing studies, AMPK phosphorylates Nrf2 at the Ser550 residue, leading to nuclear accumulation of Nrf2. AMPK also induces HO-1 production through the Nrf2/ARE pathway^42^ (59, 68), indicating that Nrf2 is a downstream signal for AMPK, both of which participate in neuroprotection following brain injury. 3-N-butylphthalate (NBP) has been shown to activate Nrf2 (69, 70). HP-1c, which is composed of telmisartan and the NBP derivative 2-(1-hydroxypentyl)-benzoic acid (HPBA), has been shown to promote M2 microglial polarization and exhibits antioxidant and anti-inflammatory effects through activation of the AMPK/Nrf2 pathway (68).

In summary, activation of AMPK and Nrf2 pathways promotes microglial M2 polarization, which reduces inflammation in the brain after stroke and plays a role in preventing further inflammation.

### Chemokine Ligand and Receptor Families

#### CX3CL1 and CX3CR1

The chemokine fractalkine (CX3CL1)/CX3CR1 receptor-ligand pair was reported to be a communication link between neurons and microglia. CX3CL1 is an inhibitory factor expressed on neurons, and its receptor, CX3CR1, is primarily expressed on microglia (13, 71–73). The combination of these two components keeps microglia quiescent. CX3CL1 release from neurons is significantly reduced in the presence of nerve injury, which leads to microglial activation (74). The CX3CL1/CX3CR1 signaling axis promotes microglial phagocytic function in the early phase after ischemia (74).

After establishing a MCAO model for local cerebral ischemia in mice, Jolivel et al. observed that serum proteins lead to pooling of activated microglia near blood vessels, and vascular endothelial cells were phagocytosed, which ultimately led to BBB disintegration. On the other hand, the CX3CR1^−/−^ (CX3CR1 loss of function) mouse model resulted in a reduction in BBB breakdown and a decrease in the area of stroke injury (12). Deficiency in CX3CR1 also leads to microglial morphological arborization and reduced expression of M1 phenotypic markers, thereby promoting microglial conversion to the M2 phenotype (74).

Overexpression of SIRT3 after ischemia leads to the upregulation of CX3CR1 expression, which promotes G protein-dependent migration of microglia (75). Thus, activation of the CX3CL1/CX3CR1 signaling axis in the early stage after ischemic stroke promotes microglial activation, migration, intrinsic phagocytosis, the release of pro-inflammatory substances, and exacerbate nerve injury. In contrast, a deficiency of the CX3CR1 receptor results in neuroprotective effects.

Recent experiments have revealed that a deficiency in CX3CR1 leads to significant reductions in the transcription factor, Nrf2 (76). These results suggest a link between CX3CR1 and Nrf2 during inflammation. CX3CL1 overexpression activates the transcription factor, Nrf2, and its target genes. For example, HO-1 limits the excessive activation of microglia, and when Nrf2 and CX3CR1 are knocked out, microglia do not express HO-1 (77). Also, CX3CL1 inhibits LPS-induced microglial activation and reduces the release of inflammatory factors in microglia, including NO, IL-6, and TNF-α through activation of the PI3k/Akt pathway, which effectively inhibits neuronal death (78, 79). The use of exogenous CX3CL1 in a MCAO mouse model reduced the local ischemia-induced cerebral infarct size, neurological deficits, and caspase-3 activation (80). These results indicate that CX3CL1/CX3CR1 signaling also plays a role in post-ischemic neuroprotection.

The studies described above demonstrate that the CX3CL1/CX3CR1 pathway can activate microglia, as well as promote their phagocytosis, migration to regions of brain tissue injury, and the release of inflammatory factors after stroke. Silencing CX3CR1 during the early post-ischemic period inhibited microglial activation, reduced the expression of inflammatory factors, polarized microglial toward the M2 phenotype, and effectively reduced the area of brain injury. Therefore, these data suggest that the lack of CX3CR1 is neuroprotective. However, the expression of CX3CR1 is not necessarily always detrimental to recovery after brain injury. Over time, the deficiency of CX3CR1 might induce other responses in the brain microenvironment, which may exacerbate brain injury in severe cases. For example, a deficiency in CX3CR1 leads to reduced expression of HO-1. Accordingly, we speculated that the functional contradiction exhibited by the CX3CL1/CX3CR1 pathway mightay be related to different times of onset as well as the course of the disease. According to recent reports, when inhibition of CX3CR1 is targeted early, or CX3CL1 expression levels are increased, administration of exogenous CX3CL1 after ischemia can reverse the neurological damage caused by stroke to some extent (81, 82). However, because this pathway contradiction results in both neuroprotective and neurodamaging effects, the specific mechanisms of action and treatment need additional, extensive research.

#### CCL2/CCR2

Monocyte chemoattractant protein-1 (MCP-1), also named CC chemokine ligand 2 (CCL2), can induce recruitment of monocytes or macrophages and increase the production of inflammatory cytokines, such as IL-1β and IL-6 (83, 84). The signaling pathway for MCP-1 is connected with CC chemokine receptor 2 (CCR2), which also plays an important regulatory role during stroke.

Overexpression of CCL2 has been shown to increase macrophage infiltration and the area of cerebral infarction at the injury site (84), suggesting that CCL2 can induce post-stroke inflammation and adversely affect post-stroke recovery. Overexpression of transforming growth factor-β1 (TGF-β1) after ischemia-reperfusion injury down-regulates CCL2, resulting in a significant reduction in the area of cerebral infarction (85), indicating that the neuroprotective effect was due to inhibition of the expression of chemokine CCL2, thus, confirming the neurotoxic effect of CCL2. Meanwhile, deficienc in CCL2 and CCR2 greatly reduce the recruitment of macrophages after stroke, the size of the cerebral infarction area, and the degree of BBB disruption after ischemia-reperfusion injury. Such deficiencies also reduce expression levels of inflammatory cytokines (86–89).

A human umbilical cord mesenchymal stem cell (HUC-MSC)-derived CCR2-overexpressing exosome, Exo^CCR2^, promotes microglial/macrophage M2-type polarization by competitively binding CCL2 to CCR2. This results in inhibition of nerve injury caused by CCL2-mediated macrophage migration and activation, reduction in the release of inflammatory factors, and decreased NF-κB expression, all of which promote microglial and macrophage M2-type polarization (90).

Therefore, activation of the CCL2/CCR2 pathway can recruit inflammatory cells and release pro-inflammatory factors, which are detrimental to the stroke prognosis. Inhibition of this pathway activity has a significant effect on ischemic stroke and cognitive impairment after stroke. Thus, targeting the inhibition of the CCL2 expression or its receptor CCR2 could improve the prognosis of stroke.

#### CXCL16/CXCR6

CXCL16 expression is increased in microglia treated with CX3CL1, and cerebral ischemia can result in overexpression of CXCL16 (91). Francesca Lepore proposed that activation of the CXCL16/CXCR6 axis regulated microglial polarization to the M2 phenotype after ischemia, and inhibited LPS- and IFNγ-mediated microglial polarization to the M1 type which, reduced the area of necrosis, promoted neuroprotective mechanisms, and inhibited ischemic neuronal death (92).

### TLR

Toll-like receptors (TLR) are signaling receptors in the innate immune system that promote microglial activation and polarization (93). Currently, 10 and 12 members of the TLR family have been found in humans and mice, respectively, and exhibit different distributions and functions. For example, TLR2, TLR4, and others are located on the cell surface, while TLR3, TLR7, TLR9, and others are located inside the cell (93). TLR can recognize both pathogen-associated molecular patterns (PAMPs) and damage-associated molecular patterns (DAMPs). The dimers that are formed bind to adaptor proteins such as MyD88, TRIF, and other chaperones to activate downstream signaling pathways, ultimately activating NF-κB and inducing the expression of inflammatory factors in microglia (94, 95).

TLR4 recognizes LPS on the cell surface and mediates microglial activation and the production of pro-inflammatory factors after brain injury (40, 96, 97). These functions aid in reducing secondary nerve injury caused by traumatic brain injury through inhibition of TLR4 signaling and regulating microglial polarization to the M2 phenotype (98).

Heme induces NF-κB activation after intracerebral hemorrhage injury by activating microglia and signaling MyD88 and TRIF through the TLR4 pathway, which in turn increases the expression of inflammatory factors and exacerbates inflammatory injury in brain tissue (99). In a model of hemorrhage stroke, the use of the TLR4 inhibitor, TAK-242, reduced the infiltration of peripheral inflammatory cells, expression of pro-inflammatory factors, and resulted in neurological deficits (100). This suggests that TLR4 is involved in the process of neuroinflammation after brain injury, and downregulation of TLR4 expression could control the adverse process to some extent. A recent study revealed that after ischemia-reperfusion treatment, smaller cerebral infarction areas occurred in TLR4 knockout mice. Also, downstream NF-κB and p65 expression were reduced, and there was no significant difference in infarct size or p65 expression levels between TLR3 and TLR9 knockout mice and wild-type mice (101).

Similarly, the use of β-caryophyllene after ischemia-reperfusion injury decreased TLR4 expression levels, reduced the release of pro-inflammatory factors, and inhibited microglial activation as well as M1-type polarization (93). These results suggest that TLR4 expression by microglia is associated with aggravation of stroke-induced secondary brain injury. Thus, targeted inhibition of TLR4 could reduce microglial activation and release of pro-inflammatory factors, coordinate the M1/M2 polarization of microglia, and play a protective role in stroke-induced brain injury.

Beside TLR4, also TLR3 plays a key role in microglia activation. TLR3 induces activation of IFN regulatory factor 3 (IRF3) and NF-κB via the TRIF pathway (96). Pan et al. established an ischemia-reperfusion model after preconditioning rats using the TLR3 agonist, polyinosinic-polycytidylic acid [poly (I:C)]. They reported that the degree of nerve injury, cerebral infarction area, and the expression levels of TNFα and IL-6 were significantly reduced compared with the control group (102). Injecting poly (I:C) into animal models after cerebral ischemia also down-regulated the conduction of the TLR4/MyD88 signaling pathway by activating the TLR3/TRIF pathway. Poly (I:C) significantly reduced the expression levels of TNF-α and IL-1β, indicating it could have a therapeutic role in ischemia-reperfusion injury (103). Based on the results discussed above, we hypothesize that there is an antagonistic effect between the TLR3 and TLR4 pathways. Activation of the TLR3/TRIF pathway could down-regulate the levels of TLR4/MyD88, reducing microglial activation and the expression of inflammatory factors to reverse the adverse effects of TLR4 on post-stroke injury. Therefore, activation of TLR3 should be investigated as a possible therapy for ischemic brain injury. In summary, TLR could be an important target for stroke treatment due to its essential role in regulating the inflammatory response.

### STAT

The JAK/STAT pathway is one of the signal transduction cascades and plays an essential role in cytokine receptor signaling (104). JAK is a member of the Janus kinase family of protein tyrosine kinases, which is found primarily on cell membranes. It binds to and phosphorylates a JAK-binding site containing domains for cytokine receptors, and subsequently forms STAT-binding sites to recruit signal transducers and activators of transcription (STATs). The STATs are then phosphorylate and translocated to the nucleus to regulate gene expression (105). IL-10 protects against inflammation by inhibiting the release of pro-inflammatory cytokines from monocytes/macrophages and the activity of LPS (106–108). One of the IL-10 signaling pathways is the JAK/STAT pathway. Binding of IL-10 to the IL-10 receptor (IL-10R) activates JAK1 and STAT3, which is necessary to induce IL-10 to inhibit macrophage activation and produce anti-inflammatory effects (109–111).

Recently, it has been reported that the neuroprotective effect induced by the inhibition of NOX may be generated through activation of the IL-10/STAT3 pathway (31). Melatonin has been shown to inhibit microglial M1-type polarization by activating STAT3 and induces M2-type polarization in microglia, which results in neuroprotection (112). Resveratrol up-regulated the expression of the suppressor of cytokine signaling 3 (SOCS3) by promoting the release of IL-10, which activated the JAK1/STAT3 signaling pathway and, in turn, inhibited LPS-induced microglial pro-inflammatory factor release and M1-type polarization (113). On the other hand, STATs also exhibit several negative roles. For example, the activation and phosphorylation of STAT1 is associated with M1 microglia activation in hypoxia-activated BV2 cells, and acts as the increased expression levels of M1 microglia (114). Furthermore, STAT3 was also associated with M1 microglia polarization in both an MCAO-induced and a bilateral common carotid arteries stenosis (BcaS)-induced model of ischemic stroke (115, 116). The results discussed above reveal the regulatory role of STAT on the inflammatory response after stroke. However, due to these contradictory evidence on regulation of microglia polarization, further studies are required.

### P2X7, P2Y12, and NLRP3

Extracellular nucleotide receptors (P2 receptors) include two families, ionotropic receptors (P2X) and metabotropic receptors (P2Y). The purinergic ligand-gated ion channel 7 receptor (P2X7) is a trimeric cation channel, which is activated by extracellular ATP. P2X7 activates Pyrin domain-containing 3 (NLRP3). After injury resulting from ischemic stroke, elevated extracellular ATP concentrations activate P2X7, triggering a series of intracellular responses that lead to NLRP3 inflammatory body activation and assembly (117). Botulinum toxin type A (BTX-A) inhibits M1-type microglial polarization and induces M2 polarization in rats through inhibition of P2X7 expression (118). A role of P2Y12 receptor in microglia activation was also shown. Indeeed, extracellular ADP acting on the P2Y12 receptor activated NF-κB and the NLRP3 inflammasome to enhance microglial inflammation (119). Furthermore, in primary cultured microglia, ticagrelor, a direct-acting, reversibly binding P2Y12-receptor antagonist, fully inhibited ADP-induced chemotaxis (120).

NLRP3 is a member of the Nod-like receptor (NLR) family (121). It is a cytosolic complex that activates caspase-1, and its interaction with the adaptor protein, Asc, also can recruit and activate caspase-1 that subsequently leads to the maturation of IL-1β and IL-18 (122, 123). It has been shown that upregulation of NLRP3 expression and increased release of pro-inflammatory cytokines in microglia after ischemia exacerbated the resulting post-ischemic neurological damage. However, after inhibiting the expression of NLRP3 in an ischemic mouse model, the brain injury caused by ischemia was alleviated by reducing the degree of cerebral infarction and the BBB disruption (124). This study also found that NLRP3 expression was down-regulated in the NOX2-deficient model, suggesting that NOX is involved in the activation and expression of NLRP3 in microglia. These results further elucidated the mechanisms by which NOX exerts its toxic effects.

Thus, P2X7, P2Y12, and NLRP3 play essential roles in regulating the occurrence of neuroinflammation after ischemic stroke. Inhibition of their expression reduces the release of pro-inflammatory factors in microglia and can alleviate post-stroke brain injury.

### GLS1

Glutaminase-1 (GLS1) is a mitochondrial enzyme that catalyzes the hydrolysis of glutamine to produce glutamate. Elevated GLS1 expression can be observed in activated microglia, and GLS1 induces inflammatory activation of microglia and the release of exosomes (125). Gao et al. observed that the degree of upregulation of GLS1 expression after ischemia was positively correlated with increased expression of microglial M1-type markers. Also, overexpression of GLS1 induced microglial activation by increasing the release of inflammatory exosomes (126). Meanwhile, inhibition of GLS1 activity by CB839 reduced the release of exosomes, which alleviated neuroinflammation resulting from cerebral ischemia. Thus, this effect was the same as inhibition of exosome release by GW4869, which also alleviated the inflammatory response (126). Therefore, inhibition of GLS1 expression might produce neuroprotective effects and could be a new treatment avenue for ischemic stroke.

### PPARγ

Peroxisome proliferator-activator receptor γ (PPARγ) is a member of the nuclear receptor family that binds to PPAR response elements (PPRE) in the promoter region of target genes. Currently three PPAR subtypes have been discovered, including PPAR-γ, PPAR-α, and PPAR-δ (127).

PPARγ is known to play a key role in regulating lipid metabolism, cell apoptosis and inflammation (128, 129). In addition, PPAR-γ activation is reported to reduce neurodegenerative and inflammatory processes in the brain (127). Recent studies have described a close relationship between PPARγ and ischemia injury, suggesting that the effect of PPARγ on ischemic injury is primarily associated with regulation of the inflammatory response (130). Also, PPARγ has been shown to coordinate the switch in the microglia/macrophage phenotype from a pro-inflammatory to an anti-inflammatory phenotype, leading to inhibition of inflammation (131). For example, a current study showed that 10-O-(N,N-dimethylaminoethyl)-ginkgolide B methanesulfonate (XQ-1H) promoted anti-inflammatory microglial polarization through activation of the PPARγ signaling pathway after ischemic stroke (130). Also, rhFGF21 treatment inhibited M1 polarization and pro-inflammatory cytokine expression in microglia by inhibiting NF-κB and upregulating PPAR-γ (132). This evidence suggests a neuroprotective effect of PPAR-γ activation on cerebral ischemic injury.

### Drug Factors Affecting Microglial Activation and Polarization

Based on the analysis described above, we conclude that microglia play a critical role in regulating the repair process that takes place in post-stroke injury. Microglia not only sense the changes in the brain microenvironment and respond through regulation of key factors but microglia, in turn, are regulated by specific signaling pathways and cytokines. Thus, finding treatments that effectively inhibit the activation and polarization of the pro-inflammatory microglial phenotype has important clinical implications for improving stroke prognosis. Some of the proven drugs categorized based on their targets are listed below (Table 1).

**Table 1:** Drug factors affecting microglial activation and polarization.

**Drug**	**Targets**	**Mechanisms**	**Therapeutic effects**	**References**
Telmisartan	AMPK	Angiotensin II receptor blocker	Promotes M2-type polarization by activating the AMPK pathway and inhibit microglial activation	(60)
HP-1c	AMPK/NRF2	A mixture of telmisartan and NBP derivatives	Activates the AMPK/NRF2 signaling pathway and regulates microglial M2-type polarization	(68)
β-caryophyllene	TLR4	Decreases TLR4 activity	Reduces the secretion of pro-inflammatory cytokines IL-1β, IL-6, and TNF-α, decreases the M1/M2 microglial ratio	(93)
TAK-242	TLR4	A TLR4 inhibitor	Reduces the expression of inflammatory factors and promotes neuroprotective effects by inhibiting M1-type polarization	(100)
poly(I:C)	TLR3	A TLR3 agonist	Reduces the expression levels of pro-inflammatory cytokines and inhibites the inflammatory activation of microglial cells	(102, 103)
Melatonin	STAT3	A hormone secreted by the pineal gland	Can cross the BBB and play a neuroprotective role in brain injury caused by ischemic stroke, regulates the polarization of microglia to the M2 phenotype through the STAT3 pathway, inhibits the neurotoxic effects of M1-type microglia, and improves neurological function	(112, 133, 134)
Resveratrol	JAK/STAT	A natural polyphenol found in a wide range of plants, an agonist of JAK/STAT pathway	Crosses the BBB, prevents ischemic brain injury, reduces the production of microglial pro-inflammatory factors, and has anti-inflammatory and neuroprotective properties, reduces the expression of pro-inflammatory cytokines IL-1β, TNF-α, and IL-6 in microglia after LPS stimulation by activating the JAK/STAT pathway and increasing the release of IL-10	(113, 135)
DBD	MAPK/NF-κB	Inhibitor of MAPK and NF-κB	Inhibits microglial activation and can reduce the release of inflammatory cytokines and up-regulate the expression of M2 markers	(136)
Anisalcohol	MAPK/NF-κB	A phenolic compound isolated from gastrodin	Inhibites LPS-induced NO production and the release of pro-inflammatory cytokines such as TNF-α in microglia, also increases the expression of factors such as TGF-β. The protective effect may be related to inhibition of the MAPK/NF-κB signaling pathway	(137)
Baicalein	MAPK/NF-κB	A bioactive ingredient extracted from the root of Scutellaria baicalensis Georgi, a NF-κB inhibit	Inhibited NF-κB activation and signaling, as well as reduced the phosphorylation of JNK, ERK, and p38, thereby reducing the release of pro-inflammatory factors IL-6, TNF-α, and others, which inhibited the polarization of microglia to the M1 phenotype	(49)
Rosiglitazone	PPAR-γ	A PPAR-γagonist	Reduces the numbers of M1 microglia and increases the numbers of M2 microglia, promotes microglial M2 polarization after MCAO in mice	(131, 138)
Malibatol A	PPAR-γ	A novel natural anti-oxidant extracted from the Chinese plant Hopea hainanensis	Decreases the infarct size and alleviates the brain injury after MCAO in mice, decreases M1 markers (CD16, CD32, and CD86) and increases M2 markers (CD206, YM-1) while promoting the activation of nuclear receptor PPARγ in MCAO mice and in LPS-stimulated microglia	(139)
XQ-1H	PPAR-γ	A novel derivative of ginkgolide B	Promotes anti-inflammatory microglia polarization via activating PPARγ signaling pathway after ischemic stroke	(130)
Montelukast	CysLT1	A potent CysLT1 receptor antagonist	Influences the phenotype of microglial cells, increasing the number of M2 polarizes microglia/macrophages, over the M1 phenotype, at acute phase after MCAO in mice	(140)
BTX-A	P2X7	Can inhibit the expression of rat P2X7	Acting as a neuroprotective agent by inhibiting M1-type microglial polarization and inducing M2 polarization	(113)
Sildenafil	PDE5	A PDE5 inhibitor	Inhibits LPS-induced M1 polarization of microglia by decreasing the production of nitric oxide, TNF-α, and IL-1β, induces M2 polarization, which has been shown to provide protection against lesion extension in the late phase of MCAo in neonatal mice	(141, 142)
Yonkenafil	PDE5	A PDE5 inhibitor	Inhibits LPS-induced M1 polarization of microglia by decreasing the production of nitric oxide, TNF-α, and IL-1β	(141)
Minocycline		A member of the tetracycline antibiotic family, a selective inhibitor of M1-type microglia	Protects against brain injury by inhibiting microglial activation and selectively inhibites microglial M1-type polarization	(35, 143)
Ginsenoside		The main active component of ginseng	Exerts anti-inflammatory effects through inhibiting the expression of pro-inflammatory cytokines and inducing M2-type polarization of microglia.	(144–147)

## Conclusion

Stroke is a leading cause of death worldwide, and effective treatments are a pressing challenge. Over the years, research into the cellular events and mechanisms associated with stroke has been ongoing. However, there are still unanswered questions, such as the activation of some targets that are protective against stroke and also have adverse effects. There are still disputes concerning how these targets can be regulated to maximize their protective effects, how to use activators of these targets, and whether the timing and dose of these drugs might have different effects on prognosis. These questions need further exploration.

It is known that microglia are rapidly activated and polarized into M1 and M2 phenotypes after stroke, which produces opposite effects on post-stroke brain injury. Based on current experiments, we conclude that inhibiting M1 microglia and promoting M2 microglia is protective against neurological damage. However, some signaling pathways and drugs have been shown to induce both M1 and M2-type polarization in microglia. Does this indicate a dual role for these pathways and drugs, or do microglia possess additional, undiscovered properties? These questions need to be resolved by further experiments. Moreover, microglia can exhibit a mixed phenotype between M1 and M2 (8). Thus, limiting microglia to the two phenotypes representing two polar states after stroke is oversimplified. Therefore, the classification and nomenclature of microglia requir further exploration.

Although successful experimental treatment protocols for stroke have been developed, they have not been entirely successful in humans. We know that the current treatment for stroke in the clinical setting is still challenging. There are two possible reasons for this: (1) In experimental models, the MCAO model is usually used to simulate stroke *in vivo* as well as the OGD model *in vitro*, which may not adequately simulate clinical stroke. This is because stroke involves a long process of disease progression, which is influenced by a range of factors, including age, gender, temperature, environment and others. Several cellular events also are involved in the stroke process. For stroke patients experiencing other diseases at the same time, the diseases may interact and could cause increases or decreases the damage caused by the stroke. This is why experimental models have limitations. (2) Species differences exist between humans and experimental models. Also, the expression level of the same targets and effects of drugs are likely to vary in different species. Therefore, experimental results can only provide therapeutic ideas for clinical research, and translational research is particularly important.

In conclusion, we suggest several recommendations: (1) Researchers should search for novel and accurate experimental stroke models. (2) The communication between human microglia and neurons should be observed directly to study the protective effects of human microglia on the nervous system. (3) Stroke-related pathway proteins, targets or markers of M1 and M2 microglia phenotypes should be extracted and evaluated from the cerebrospinal fluid or serum of stroke patients to observe the effects of stroke on different molecular pathways and cell phenotypes in humans. (4) Additional drugs need to be identified that may have protective effects against stroke, e.g., network pharmacology should be used to find herbal medicines that have the same targets as stroke, and identify new nanomaterials. In addition, the mechanisms that influence microglia activation and polarization during stroke need to be studied in greater depth.

## Author Contributions

RD and JW: conceptualization. RD: writing—original draft preparation. JW, RH, and HL: writing—review and editing. ZX: reading and suggestions. All authors have read and agreed to the published version of the manuscript.

## Conflict of Interest

The authors declare that the research was conducted in the absence of any commercial or financial relationships that could be construed as a potential conflict of interest.

## Abbreviations

CNS: Central nervous system
NO: Nitric oxide
BBB: Blood-brain barrier
MCAO: Middle Cerebral Artery Occlusion
ROS: Reactive oxygen species
Aβ: Amyloid-β peptide
NF-κB: Nuclear factor kappa-B
NGF: Nerve growth factor
NOX: NADPH oxidase
TNF-α: Tumor necrosis factor-α
IL-6: Interleukin 6
II-1β: Interleukin 1β
IL-10: Interleukin-10
TGF-β: Transforming growth factor-β
LPS: Lipopolysaccharide
LCN2: Lipocalin 2
VEGF: Vascular endothelial growth factor
AMPK: AMP-activated protein kinase
Nrf2: Nuclear factor erythroid-2-related factor 2
LKB1: Liver kinase B1
CaMKKβ: Calmodulin-dependent protein kinase kinase β
Keap1: Kelch-like ECH-associated protein 1
ARE: Antioxidant response elements
HO-1: Heme oxygenase-1
NQO-1: NADPH quinone oxidoreductase-1
GST: Glutathione S-transferase
ATF4: Activating transcription factor 4
MCP-1: Monocyte chemoattractant protein-1
CCL2: CC chemokine ligand 2
CCR2: CC chemokine receptor 2
HUC-MSC: Human umbilical cord mesenchymal stem cell
TLR: Toll-like receptors
STAT, Signal PAMP: Pathogen-associated molecular pattern
DAMP: Damage-associated molecular patterns
STAT: Signal transducers and activator of transcription
SOCS3: Suppressor of cytokine signaling 3
PPAR-γ: Peroxisome proliferator-activator receptor γ
BTX-A: Botulinum toxin type A
NLR: Nod-like receptor
DBD, 3: 4-Dihydroxybenzaldehyde
GLS1: Glutaminase-1
BTX-A: Botulinum toxin type A
PDE5: phosphodiesterase of type 5
ARB: Angiotensin II receptor blocker.
